# Leukocyte-epithelial physical contacts mediate interstitial migration in vivo

**DOI:** 10.21203/rs.3.rs-8594440/v1

**Published:** 2026-02-05

**Authors:** Jonathan H. Schrope, Tanner F Robertson, Adam Horn, Jack J. Stevens, Clyde W. Tinnen, Julie Rindy, Yiran Hou, Emilie Rochon, David J. Beebe, Anna Huttenlocher

**Affiliations:** 1 Department of Biomedical Engineering, University of Wisconsin-Madison, Madison, WI, USA.; 2 Department of Medical Microbiology and Immunology, University of Wisconsin-Madison, Madison, WI, USA.; 3 Department of Pathology and Laboratory Medicine, University of Wisconsin-Madison, Madison, WI, USA.; 4 Medical Scientist Training Program, University of Wisconsin-Madison, Madison, WI, USA.; 5 Department of Pediatrics, University of Wisconsin-Madison, Madison, WI, USA.; 6 Carbone Cancer Center, University of Wisconsin-Madison, Madison, WI, USA.

## Abstract

Efficient immune cell migration requires physical interactions with surrounding tissues. While tissue matrix mechanics influence leukocyte motility, it is unknown how leukocytes exert pushing and pulling forces to traverse tightly adherent epithelial tissues, which comprise a majority of tissue volume in vivo. Here, we leverage the optical transparency of larval zebrafish to identify how physical interactions with epithelial cells regulate mechanisms of neutrophil force generation to navigate cell-dense tissues. Confining forces from epithelial cells induce a mechanosensitive central actin network, mediated by Cdc42 and WASP, which exerts expansile forces on surrounding cells to dilate a path for migration. In concert, direct cell-to-cell (leukocyte-epithelial) contacts, mediated by integrin ɑE binding to epithelial cadherin, generate tractional forces to enable forward motility. Together, our findings identify how physical interactions with surrounding epithelial cells regulate leukocyte motility through cell-dense tissues in vivo.

Effective immune responses require leukocytes to traverse complex tissue environments. This requires generation of both expansile forces to overcome tissue resistance, and tractional forces which couple actomyosin machinery to the environment^1–5^. Together, these processes drive forward motion. Mechanisms of force transmission between leukocytes and matrix tissue have been well characterized, largely by leveraging in vitro models. Actin polymerization at the cell front, regulated by Rho family GTPase activation of actin nucleation proteins Wiskott Aldrich Syndrome Protein (WASP) and WASP family verprolin-homologous protein (WAVE), push the membrane forward along the axis of motility^2,6^. To overcome mechanical resistance provided by matrix tissue in vitro, dendritic cells and T cells utilize Dedicator of Cytokinesis 8 (DOCK8)-mediated actin polymerization to push outward against matrix components to dilate a path for the cell nucleus^7,8^. Forward motility along this path requires coupling intracellular forces to the environment via traction forces, the mechanisms of which depend on surrounding mechanical context. Membrane spanning integrins physically link actomyosin machinery to matrix tissues in the form of adhesive complexes^9^. However, under confinement, cells can migrate independent of integrins^10^, instead reliant on frictional forces at the cell-substrate interface^11^ or aberrations in environmental topology to propel their way forward^12^. While well established that matrix properties regulate mechanisms of cell motility, the majority of tissue volume is composed of cell-dense environments, which offer significantly different mechanical properties than matrix tissues^13^ and lack matrix components critical for integrin-mediated tractional interactions. Therefore, it remains unclear how leukocytes generate expansile and tractional forces to navigate adherent epithelial cell environments in situ.

To address these questions, we leverage the optical access afforded by the larval zebrafish to capture neutrophil-epithelial interactions during interstitial migration at single cell resolution using real time imaging. We discover differences in actin dynamics between neutrophils migrating through matrix-rich tissue, versus the cell-dense epithelium where neutrophils physically interact with surrounding cells. We find that neutrophils deploy a mechanosensitive, centralized F-actin network, mediated by the Rho-GTPase Cdc42 and its effector WASP, to overcome epithelial cell resistance and dilate a space for migration. At the same time, focal actin networks at the cell edge are regulated by direct physical interaction with epithelial cells mediated by integrin ɑE binding epithelial cadherin (E-cad), indicating a cell-on-cell (leukocyte-epithelial) mechanism of migration. In all, this work identifies how interactions with surrounding cells regulate leukocyte actin dynamics to enable motility through cell-dense tissues in vivo.

## Cell-dense epithelial maze constrains leukocyte motility

**Fig. 1:**
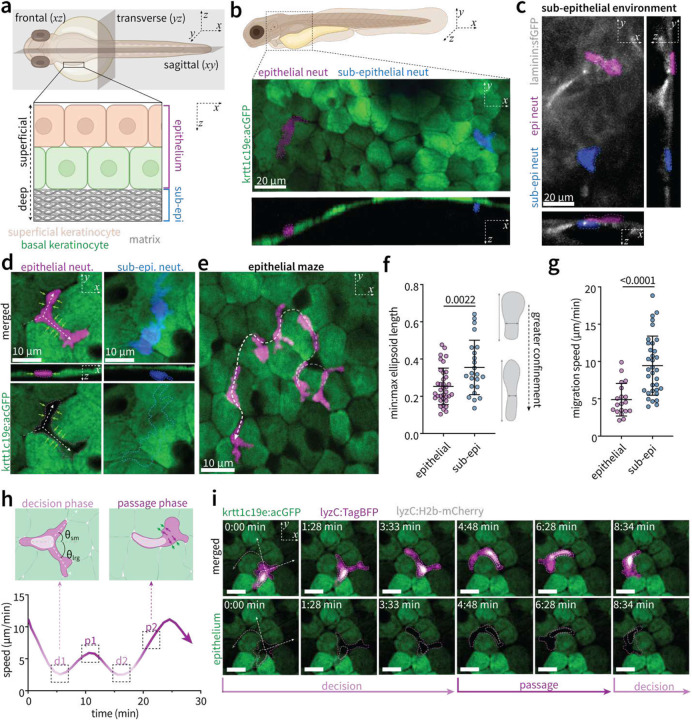
Neutrophils physically interact with surrounding cells to navigate an epithelial maze **a)** Schematic of zebrafish skin at 3 days post-fertilization (dpf), composed of a two-cell thick epithelial layer of superficial (tan) and basal (green) keratinocytes and a deeper, matrix-rich sub-epithelial layer. **b)** Multi-view maximum intensity projections of neutrophils (mpx:mCherry) within the epithelium (pseudocolored magenta) and sub-epithelial layer (pseudocolored blue). **c)** Multi-view maximum intensity projections of epithelial neutrophils (pseudocolored magenta) and sub-epithelial neutrophils (pseudocolored blue) neutrophils) located superficial to, or embedded directly within, laminin matrix tissue (lamc1:lamc1-sfGFP, grey). **d)** Multi-view maximum intensity projections of neutrophils migrating within the epithelial and sub-epithelial layers. Dashed white arrows indicate potential migration paths; green arrows depict confining forces from surrounding epithelial cells. **e)** Maximum intensity z-stack, time projection depicting neutrophil motility through the epithelium. Dashed white line denotes migration path. **f)** Quantification of neutrophil confinement defined by the ratio of maximum to minimum ellipsoid axes derived from three-dimensional confocal reconstructions of individual cells in the epithelial (n=36) and sub-epithelial compartments (n=26) pooled over three independent experiments. Schematic depicts the two-dimensional corollary of the measurement (aspect ratio). **g)** Quantification of migration speed of individual cells within the epithelial (n=21) and sub-epithelial (n=34) layers, pooled over three independent experiments. **h)** Schematic and representative plot of intra-epithelial migration speed throughout distinct phases of decision-making and confined passage along the path of least deflection (lesser !). Purple arrows depict neutrophil expansile forces to overcome confining forces (green arrows) asserted by epithelial cells. **i)** Maximum intensity z-stack images depicting nuclear positioning throughout the decision-making and passage phases during intra-epithelial motility. Dashed white arrows depict angles of potential migration paths; grey dashed line indicates chosen migration direction. For **f** and **g**, *P*-value determined by two-tailed *t*-test; error bars depict standard deviation.

To understand how environmental tissue context regulates mechanisms of leukocyte motility, we performed timelapse imaging of neutrophil migration within distinct compartments of the larval zebrafish skin (Fig. 1a). We find that neutrophils spontaneously inhabit both the cellularly dense epithelial layer and a cell-sparse, matrix-rich sub-epithelial layer (Fig. 1b–c, **Extended Data Fig. 1, Supplementary Video 1**). Within the epithelium, neutrophils directly interact with surrounding cells, which mechanically confine neutrophils and act as physical determinants of migration path (Fig. 1d). The result is an epithelial “maze” (Fig. 1e, **Supplementary Video 2**), whereby mechanical resistance provided by epithelial cells results in greater neutrophil confinement (Fig. 1f) and reduced migration speed (Fig. 1g) compared to the cell-sparse sub-epithelial layer.

Efficient intra-epithelial migration therefore requires neutrophils to navigate cellular barriers by electing paths of least resistance and exerting expansile forces to deform surrounding epithelial cells to generate space for migration. There is growing interest in how leukocytes make directional decisions to circumvent mechanical barriers. In microfluidic mazes, cells can locally degrade global chemoattractant to resolve directional decisions and avoid “dead ends”^14^. Furthermore, when potential paths exhibit differences in hydraulic resistance (pre-determined by microfluidic channel cross-sectional area or matrix pore size), leukocytes elect the path of lesser resistance^15^, sensed through forward positioning of the nucleus^16^. In cell-packed epithelial tissues, devoid of clear “dead ends” or differences in hydraulic resistance characteristic of matrix tissues, we find that neutrophils elect the path requiring lesser reorientation of the cell body relative to the incoming path, pre-defined by epithelial architecture (**Extended Data Fig. 2a-b**). This manifests as a slow decision-making step whereby neutrophils extend protrusions down potential paths, followed by rapid passage directly between epithelial cells (Fig. 1h). The nucleus remains at the cell rear during both steps, with rearward positioning accentuated during periods of confined passage between epithelial cells (Fig. 1i, **Fig. S2c-d, Supplementary Video 3**). These data indicate that surrounding cells act as physical barriers which constrain motility through cell-dense tissues. Efficient passage therefore requires generation of expansile forces to deform surrounding cells (**Extended Data Fig. 2e**) and make space for the nucleus-containing cell body (**Supplementary Video 4**), coupled with tractional forces that pull the cell forward, which we explore in the following sections.

## Confinement by surrounding epithelial cells elicits a central actin burst

**Fig. 2:**
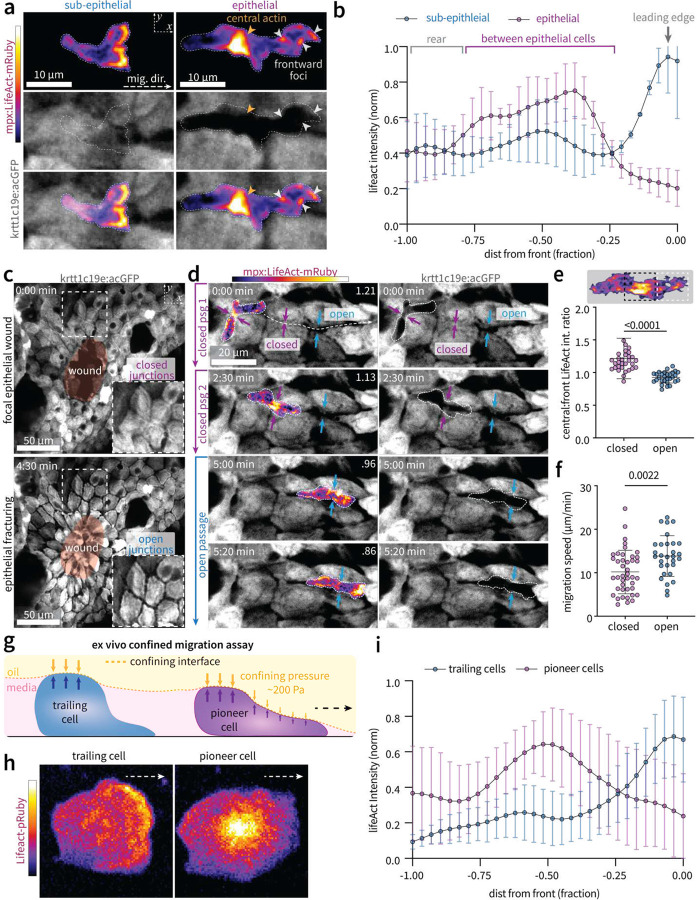
Mechanical confinement evokes a centralized actin network. **a)** Maximum intensity z-stack projections of LifeAct signal during sub-epithelial migration (left) and intra-epithelial passage (right). Yellow and white arrows indicate central actin network and frontward actin foci, respectively. **b)** Line profiles representing normalized spatial distributions of neutrophil LifeAct intensity during migration within the sub-epithelial and epithelial layers. Error bars represent standard deviation of n=6 cells pooled equally over three independent experiments. **c)** Maximum-intensity z-stack projection images depicting tissue fracturing following laser wounding, characterized by increased space between epithelial-epithelial contacts defined as either “closed” (magenta) or “open” (blue) junctions **d)** Maximum intensity z-stack projection images depicting LifeAct spatial localization during passage between two closed junctions (magenta arrows, top two images) and one open junction (blue arrows, bottom two images). **e-f)** Quantification of the ratio between LifeAct intensity at the center third (black dashed box) compared to front third (white dashed box) of the cell (e), and migration speed (f), during passage through closed or open epithelial junctions. Each data point represents the mean of a single cell (n = 21 closed and n = 24 open for e; n = 42 closed and n = 30 open for f), pooled over three independent experiments; *P*-value determined by unpaired t-test assuming equal variance. **g)** Schematic of in vitro confined migration assay depicting interaction of pioneer and trailing cells with the liquid-liquid interface. **h)** Representative images of LifeAct intensity in pioneer and trailing PLB-985 neutrophil-like cells. **i)** Line profiles representing LifeAct intensities of trailing (low confinement) and pioneer (high confinement) PLB-985 cells. Error bars represent standard deviation cells (n=9) pooled equally over three independent experiments. For **e** and **f**, *P*-value determined by two-tailed; error bars indicate standard deviation.

To investigate how leukocytes exert expansile and tractional forces to transit through adherent epidermal cells, we compared spatiotemporal F-actin dynamics in neutrophils migrating through the cell-rich epithelium and the matrix-rich sub-epithelium. In the sub-epithelium, neutrophil F-actin polymerization, visualized via LifeAct-mRuby, broadly localized to the leading edge (Fig. 2a, **left**), consistent with expansile forces generating a leading-edge pseudopod. During direct passage between epithelial cells, however, two distinct LifeAct domains emerged: 1) a population of centralized actin, and 2) distinct foci localized to the cell front (Fig. 2a, **right**). Given the fundamental role of F-actin in generating forces required for motility, these data indicate distinct migration strategies dependent on environmental context. We therefore sought to investigate the underlying mechanisms and functional role of these distinct F-actin populations observed during intra-epithelial migration, starting with the central F-actin network.

We hypothesized that central actin is only identified in the cell-rich epithelium because it is mechanosensitive, induced by confining forces exerted by surrounding cells (Fig. 2b). To test this hypothesis, we modulated the degree of confinement applied by epithelial cells and measured spatial LifeAct intensity distribution across the cell length. Specifically, we leveraged a recent observation that zebrafish wounding induces epithelial tissue “fracturing”^17^, which increases the space between epithelial cells. Focal laser wounding of the epithelium generated a zone of fractured tissue, characterized by increased space between epithelial cells which are defined as “open junctions” (Fig. 2c) and a more distant zone of “closed junctions” (**Extended Data Fig. 4a**). We quantified the spatial distribution of neutrophil LifeAct intensity during passage between either “open” or “closed” junctions (defined as greater than or less than 1 μm between epithelial cells, see **Extended Data Fig. 4b**). Passage through open junctions resulted in the spatial redistribution of F-actin from the cell center to the leading edge (Fig. 2d,e) and faster migration (Fig. 2f). To rule out the possibility that chemical cues released during wounding influenced observed differences in F-actin distribution, we examined LifeAct dynamics in human-derived, neutrophil-like PLB-985 cells under confinement by a soft, liquid-liquid interface that we previously showed replicates the forces applied by surrounding cells during intra-epithelial migration^18^. In this system, the first cells that contact the interface, defined as pioneer cells, migrate under confining forces similar to those of “closed” epithelial junctions, succeeded by follower cells that migrate under reduced mechanical confinement^18^ (Fig 2g). Pioneer cells exhibited a similar central actin network to that observed during intra-epithelial migration in vivo, while follower cells localized their F-actin to the leading edge (Fig. 2h–i). Collectively, these data support a model whereby formation of the central actin network is dependent on confining forces applied by surrounding epithelial cells. We next sought to investigate the functional relevance and underlying molecular regulation of the central actin network.

## A mechanosensitive central actin network mediated by Cdc42 and WASP exerts expansile forces to deform surrounding cells

**Fig. 3:**
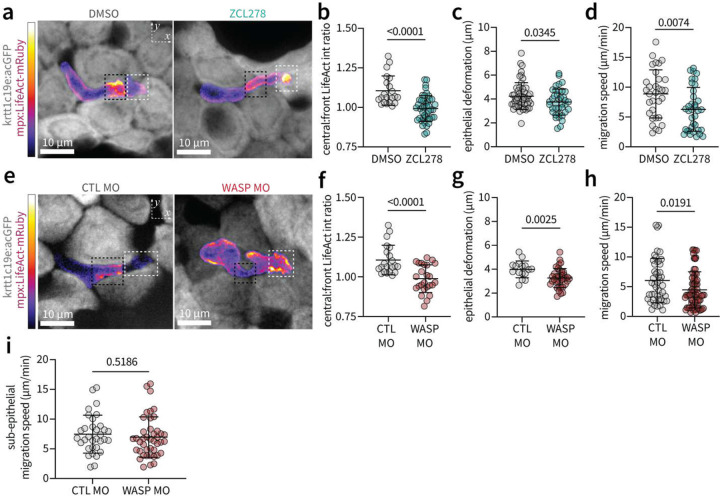
The centralised actin network is mediated by Cdc42 and WASP. **a)** Maximum intensity z-stack projections depicting spatial localization of LifeAct intensity under inhibition of Cdc42 by ZCL278. Black dashed box indicates central actin, white dashed box denotes leading edge actin. **b)** Quantification of the intensity ratio between central and leading edge LifeAct signal in single cells under treatment with DMSO (control, n=24) or ZCL278 (n=44) **c)** Quantification of maximum epithelial deformations during neutrophil passage events under treatment with DMSO (control, n=24) or ZCL278 (n=38) **d**) Quantification of migration speed of individual neutrophils under treatment with DMSO (control, n=32) or ZCL278 (n=36). **e)** Maximum intensity z-stack projections depicting spatial localization of LifeAct intensity under morpholino-mediated knockdown of WASP1. **f**) Quantification of the intensity ratio between central and leading edge LifeAct signal within individual neutrophils following injection with control (n=16) (CTL) or WASP (n=25) morpholino (MO). **g)** Quantification of maximum epithelial deformations during neutrophil passage events following injection with CTL MO (n=16) or WASP MO (n=25). **h**) Quantification of migration speed of individual cells following injection with CTL MO (n=47) or WASP MO (n=69). In all plots, *P*-value was determined by two-tailed *t*-test assuming equal variance, with error bars depicting standard deviation of datapoints pooled over at least three independent experimental replicates.

Given its spatial positioning in front of the nuclear-containing cell body, we hypothesized that the central F-actin network exerts expansile forces to deform surrounding epithelial cells and generate space for migration. Recent in vitro studies have implicated a role of the Rho-GTPase Cdc42^7,8^ and its effector WASP^8,19^ in regulating confined leukocyte migration through matrix tissue. To investigate a mechanosensitive role of Cdc42-WASP signaling in generating expansile forces during intra-epithelial motility in vivo, we quantified LifeAct spatial localisation under chemical inhibition of Cdc42 and morpholino-based knockdown of WASP^20,21^. Inhibition of Cdc42 resulted in spatial redistribution of LifeAct to the leading edge (Fig. 3a,b), decreased outward deformation of surrounding epithelial cells (Fig. 3c, **Extended Data Fig. 5a**) and impaired motility (Fig. 3d).

Knockdown of the Cdc42 effector WASP resulted in a similar F-actin redistribution (Fig. 3e,f), decreased epithelial deformations (Fig. 3g, **Extended Data Fig. 5b**), and impaired neutrophil motility (Fig. 3h). Importantly, depletion of WASP did not affect motility within the matrix-rich sub-epithelial layer (Fig. 3i). This indicates a specific role of WASP in generating expansile forces via formation of a central F-actin network to deform surrounding cells during neutrophil intra-epithelial motility in vivo. We next turned our attention to how neutrophils couple intracellular actin machinery to the cell-dense environment to generate tractional forces during intra-epithelial motility.

## Neutrophils utilize direct interactions with epithelial cells to generate tractional forces

**Fig. 4:**
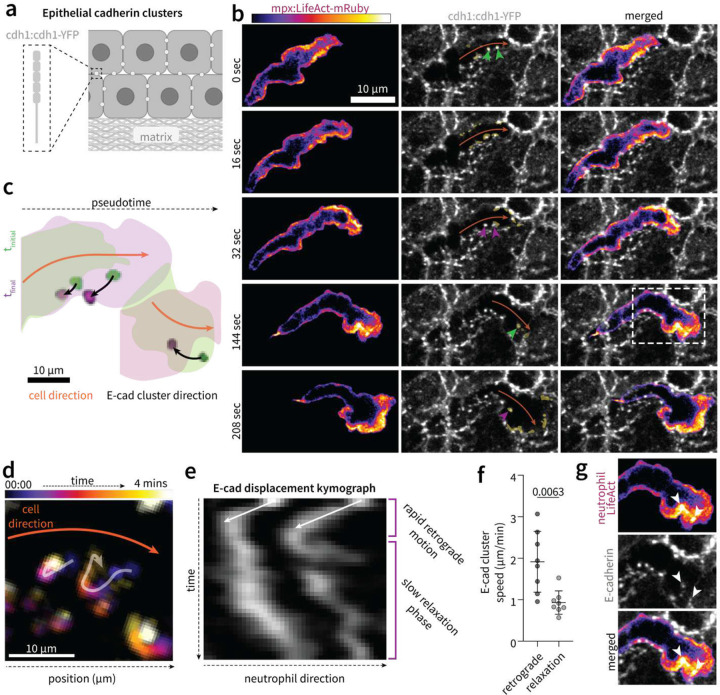
Retrograde motion of epithelial cadherin clusters during intra-epithelial neutrophil motility. **a)** Schematic depicting labeling of epithelial cadherin (E-cad) clusters (cdh1:cdh1-YFP) **b)** Maximum intensity z-stack projections depicting neutrophil F-actin dynamics (mpx:LifeAct-mRuby) and E-cadherin (grey) cluster displacements relative to neutrophil direction (orange arrow) during passage between epithelial cells. Green and magenta arrows depict initial and final time points of E-cad cluster positions, respectively. Dashed white box indicates insert corresponding to panel g. **c)** E-cad displacements (black arrows) overlaid with schematics representing neutrophil motion (orange arrow); depicting two distinct events along the same path of migration, where green corresponds to initial timepoint and magenta a later timepoint corresponding to maximal E-cad cluster displacement. **d-e**) Representative time-projection image (d) and kymograph (e) depicting retrograde E-cadherin cluster motion during neutrophil intra-epithelial passage. **f)** Quantification of E-cadherin cluster velocity during neutrophil intra-epithelial passage, depicting phases of retrograde motion and relaxation. Each data point represents mean E-cad cluster velocities (n>2) of individual passage events (n=8), pooled over three independent experiments. *P*-value determined by two-tailed *t*-test with error bars depicting standard deviation. **g)** Representative images depicting neutrophil LifeAct local maxima (white arrows) co-localization with epithelial cadherin clusters (grey).

Cell motility requires tractional forces which couple intracellular actin machinery to the extracellular environment, typically through integrin-mediated cell adhesion to matrix components. The observation that neutrophils directly interact with surrounding cells during intra-epithelial migration (**Supplementary Video 3–4**), led us to hypothesize that neutrophils might utilize direct cell-to-cell (leukocyte-epithelial) contacts to generate tractional forces. Quantification of traction forces in vitro is typically accomplished by measuring displacement of fluorescent markers embedded in elastic matrices of known mechanical properties. Recent work has quantified tractional forces between zebrafish lateral line primordial cells and matrix tissue in vivo by tracking the displacement of photobleached regions of the underlying basement membrane tissue^22^. To test our hypothesis of direct cell-on-cell migration, we leveraged the fact that epithelial cadherin (E-cad) natively organizes into distinct clusters along epithelial cell membranes in the larval zebrafish^23–26^. These membrane-bound clusters, labeled by endogenously tagged E-cadherin (Cdh1:Cdh1-YFP)^23^ (Fig. 4a), exhibit minimal displacements over timescales relevant to neutrophil migration (**Extended Data Fig. 6, Supplementary Video 6**) and therefore serve as surrogate markers capable of resolving force directionality at the leukocyte-epithelial interface. If surrounding epithelial cells act as passive mechanical barriers, E-cad clusters should move antiparallel to the migration axis. However, E-cad clusters instead exhibited rapid retrograde motion relative to the direction of neutrophil migration (Fig. 4b–c, **Supplementary Video 7**). This was followed by a slow, relaxation phase following neutrophil passage (Fig. 4e–f). This supports a model whereby active tractional forces are exerted onto epithelial cells, followed by passive relaxation during passage of the neutrophil cell body. In addition to retrograde motion indicative of neutrophil-epithelial (cell-on-cell) tractional interactions, we find that local maxima of LifeAct intensity at the leading edge co-localized with E-cad clusters (Fig. 4g). This is compatible with direct tractional interactions with E-cadherin regulating leading edge actin dynamics.

## Integrin ɑE-mediated interaction with epithelial cadherin regulates frontward actin foci to enable intra-epithelial migration

Having linked leukocyte-epithelial interactions to the generation of tractional forces, we sought to uncover the molecular mechanism which couples these interactions to the regulation of intracellular actin. Some epithelial-localized leukocytes cells express the ɑEβ7 integrin (CD103), the ligand for which is E-cadherin,^27,28^ which controls their retention within these sites^29^. However, a direct mechanistic role of ɑEβ7 integrin in regulating leukocyte interstitial migration is yet to be identified. Given spatial co-localisation of actin foci with E-cad clusters, we hypothesized that integrin ɑE-mediated interaction with E-cad generates tractional forces to mediate intra-epithelial neutrophil motility.

**Fig. 5:**
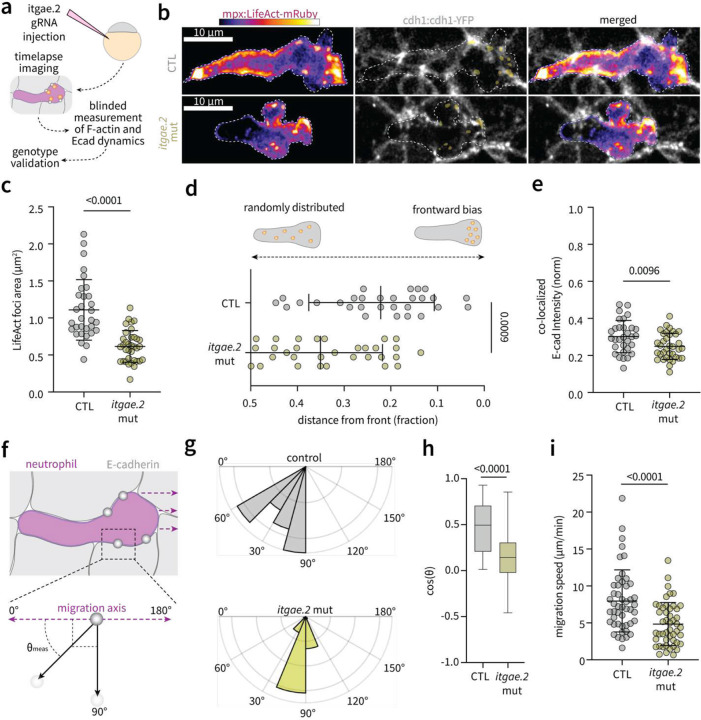
Leading edge F-actin foci are regulated by ɑE integrin-E-cadherin interactions with surrounding cells. **a)** Schematic depicting analysis of actin and E-cad dynamics of CRISPR-generated *itgae.2* mutant larval fish. **b)** Representative images of neutrophil LifeAct foci and surrounding E-cad clusters (cdh1:cdh1-YFP; grey) during intra-epithelial passage events. **c)** Quantification of LifeAct cluster size in control (tyrosinase gRNA injection) (n=35) versus *itgae.2* mutant (n=35) zebrafish. **d**) Quantification of LifeAct foci spatial distribution (defined as distance from the leading edge) in control (n=31) versus mutant (n=35) zebrafish. **e)** Quantification of E-cadherin signal intensity co-localized with F-actin foci, normalized to maximum E-cad intensity within each passage event, in control (n=31) and mutant (n=35) fish. **f)** Schematic depicting measurement of E-cad cluster motion relative to neutrophil migration direction during intra-epithelial passage. **g**) Rose plots depicting E-cad displacement angles with respect to neutrophil migration direction in control (n=35) and *itgae.2* mutant (n=53) fish. **h)** Quantification of E-cad cluster displacement angles represented as cos(*θ*). **i)** Migration speed of control versus *itgae.2* mutant zebrafish. For **c**,**d**,**e**,**h**,**i**, Error bars denote standard deviation; datapoints indicate means of individual cells pooled over three independent experiments; statistical significance determined by two-tailed t-test assuming equal variance.

To test this, we quantified LifeAct and E-cad dynamics following CRISPR-mediated interference of ɑE integrin of the aEb7 integrin complex (Fig. 5a–b). While zebrafish have two copies of ɑE integrin, (*itgae.1* and *itgae.2*), single cell analysis shows that neutrophils do not express *itgae.1* (**Fig. S7**). We therefore injected larvae expressing mpx:LifeAct-mRuby and cdh1:cdh1-YFP with guide RNA’s targeting a 5,500 base pair region of *itgae*.2 (13,006 bp total) and analyzed F-actin and E-cad dynamics in a blinded fashion, later linking measurements to genotype (**Extended Data Fig. 8a-b**). Depletion of *itgae.2* resulted in decreased size of F-actin foci (Fig. 5c), impaired F-actin foci localisation to the cell front (Fig. 5d) and decreased co-localization of E-cadherin signal with LifeAct local maxima (Fig. 5e). Concurrently, depletion of *itgae.2* mitigated retrograde motion of E-cad clusters (Fig. 5f–h) and impaired neutrophil migration speed through the epithelium (Fig. 5i). These data indicate that ɑE integrin mediates neutrophil traction forces through recognition of epithelial cadherin to regulate F-actin dynamics at the cell front during interstitial migration. Importantly, ɑE integrin does not regulate central actin pushing forces (**Extended Data Fig. 8**), indicating independent mechanisms of expansile and tractional force generation during intra-epithelial motility. These mechanisms, both regulated by physical contacts with surrounding cells, converge to regulate actin dynamics.

## Conclusions

**Fig. 6:**
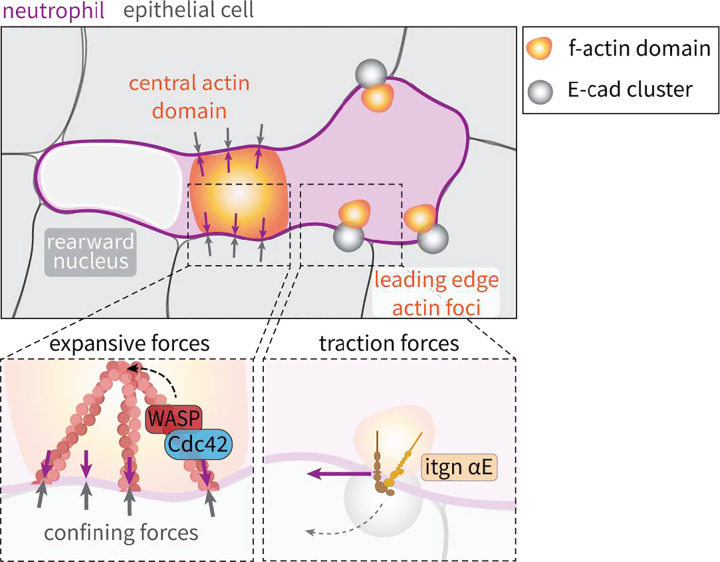
Schematic depicting how physical interactions with surrounding cells regulate actin networks and force generation during intra-epithelial motility.

In light of decades of work elucidating how migrating cells exert expansile and tractional forces onto matrix tissues, how cells generate forces to migrate through tightly adhered epithelial cells is unclear. Here, we show that confined amoeboid motility in epithelial tissues is mediated by distinct F-actin networks that transmit both pushing and pulling forces onto surrounding cells to facilitate intra-epithelial motility in a live animal. Pushing forces, in response to confining epithelial cell interactions, are generated by a Cdc42 and WASP-mediated central actin network that displaces surrounding cells to generate space for the nucleus-containing cell rear. This mechanism is not employed during more rapid neutrophil motility through the matrix-rich sub-epithelial tissues, or after epithelial tissue fracturing induced by wounding, suggesting an ability of leukocytes to dynamically adopt distinct mechanisms of actin regulation in situ in a context-dependent manner. Pulling forces, exerted directly onto epithelial cells, are generated through ɑE integrin-dependent interactions with E-cadherin which regulate actin foci at the cell front. These distinct pathways, both dependent on physical contacts with surrounding cells, control the spatial distribution and function of F-actin networks within migratory neutrophils in situ (Fig. 6).

Previous studies have demonstrated integrin-independent leukocyte motility under confinement, both in vitro and within the murine lymph node^10^. The transition to integrin-independent motility is thought to be driven by a high confinement, low adhesion environment^30^. While epithelial tissue has traditionally been defined as a low adhesion environment for leukocytes (similar to lymph node tissue), our findings uncover tractional interactions via ɑE integrin recognition of E-cadherin. Where ɑE integrin is expressed by a number of leukocytes, our data represent the first evidence of its direct role in facilitating intra-epithelial leukocyte migration. This finding indicates that within confined environments in situ, tissue context (i.e. cell type/surface expression) plays an understated role in regulating leukocyte motility.

Only recently have groups begun to expand upon the canonical paradigm of cell-on-matrix migration to explore how surrounding cells serve as living substrates to regulate mechanisms of cell motility^31–37^. We postulate that the mechanisms of actin regulation outlined here likely regulate cell-on-cell migration in a variety of biological systems including early developmental processes to human disease contexts. For example, the vast majority of tissue volume in the human body is composed of cells, which offer significantly different mechanical properties than matrix fibers (i.e. ~Pa vs ~Gpa stiffness)^13^. Therefore, many disease states are the result of dysregulated leukocyte motility through cell-dense epithelial tissues. The development of therapeutic strategies to modulate the immune system in the context of infection, malignancy, or autoimmune disease therefore requires an understanding of how leukocytes traverse cell-dense tissues. This study lays the groundwork for such an understanding, by identifying mechanisms of leukocyte force generation enabled through physical contacts with the cellular environment during intra-epithelial migration in situ.

## Methods

### Zebrafish maintenance and handling

Animal care and use was approved by the Institutional Animal Care and Use Committee of University of Wisconsin and strictly followed guidelines set by the federal Health Research Extension Act and the Public Health Service Policy on the Humane Care and Use of Laboratory Animal, administered by the National Institutes of Health Office of Laboratory Animal Welfare. All protocols using zebrafish in this study were approved by the University of Wisconsin-Madison Research Animals Resource Center (protocol M005405-A02). All transgenic lines including *Tg(mpx:mCherry)*^38^, *Tg(LyzC:TagBFP)*^39^*, Tg(LyzC:H2B-mCherry)*^40^, *Tg(mpx:LifeAct-mRuby)*^38^, *Tg(krtt1c19e:acGFP)*^41^ *(gift from Alvaro Sagasti lab), TgBac(Lamc1:Lamc1-sfGFP)*^22^ (gift from Knaut lab), *Tg(Cdh1:Cdh1-YFP)*^23^ (gift from Tobin lab), were maintained on the AB background strain. When necessary, larvae were screened for positive fluorescence using a Zeiss Zoomscope EMS3/SyCoP3 with a Plan-NeoFluar Z objective. Following breeding, fertilized embryos were transferred to E3 medium (5 mM NaCl, 0.17 mM KCl, 0.44 mM CaCl_2_, 0.33 mM MgSO_4_, 0.025 mM NaOH, and 0.0003% Methylene Blue) and maintained at 28.5°C. Larval zebrafish were anesthetized using 0.2 mg/ml tricaine (ethyl 3-aminobenzoate; Sigma-Aldrich) before any experimentation or live imaging.

### Live imaging and morphological analysis

To stabilize fish for live imaging, larvae were mounted in 1% low-melting point agarose (Sigma-Aldrich) on a 35 mm glass bottom dish (CellVis). Live imaging of zebrafish was performed using a Nikon Eclipse Ti2 microscope equipped with a Crest X-light V3531 spinning disc unit and a Kinetix22 monochrome camera. Images were acquired using either Plan Apochromat S Fluor 40X/1.30 NA, or Plan Apochromat 60X/1.42 NA objectives. Three dimensional reconstructions (Extended Data Fig. 1e) and subsequent morphology measurements (Fig. 1f) were generated in Imaris 11. Migration speed through the epithelial maze and sub-epithelium (Fig. 1g) was quantified via the FIJI/ImageJ Manual Tracking plugin on z-stack projection, timelapse movies taken at 10–20 second intervals.

### Larval zebrafish fixation and staining

To characterize differences in cellularity and matrix composition between the epithelial and sub-epithelial compartment (Extended Data Fig. 1), zebrafish larvae (*TgBac(Lamc1:Lamc1-sfGFP) or Tg(krtt1c19e:acGFP))* were fixed overnight at 4°C in 1.5% formaldehyde (Polysciences, Warrington, PA) prepared in a solution containing 0.1 M Pipes (Sigma-Aldrich), 1.0 mM MgSO_4_ (Sigma-Aldrich), and 2 mM EGTA (Sigma-Aldrich). The following day, samples were washed three times with .1% Tween-20 in PBS and permeabilized for one hour in 2% Triton X-100 at room temperature on a shaker plate. Samples were then stained with either Hoechst 33342 (10μg/mL; Invitrogen, H1399) to label cell nuclei or Alexa Fluor 594-conjugated phalloidin (1:50 dilution of stock, Sigma Aldrich A12381) to label F-actin, in 2% Triton X-100 for one hour. Samples were then washed three times with .1% Tween-20 in PBS and stored at 4° prior to imaging. Z-stacks were obtained on a Nikon Eclipse Ti2 confocal microscope at 200nm step size with the 60X objective (see above for details).

#### Nuclear positioning during intra-epithelial motility

*Tg(LyzC:TagBFP)* fish were crossed with *Tg(LyzC:H2B-mCherry)* fish to generate a stable, dual-colored zebrafish line labeling neutrophil nuclei and cytoplasm with mCherry and BFP, respectively. These fish were then crossed with *Tg(krtt1c19e:acGFP)* fish to label basal keratinocytes. Timalepase movies of neutrophil intra-epithelial motility were acquired at frame rates of 10–20 seconds. Nuclear width and positioning (Extended Data Fig. 2) were quantified manually in FIJI/ImageJ. Nuclear positioning was defined as the distance distance between the front of the nucleus to the cell leading edge, given as a rati owith respect to the total cell length.

### Quantifying actin spatial distribution and epithelial deformations

Line profiles of LifeAct intensity across the length of migrating neutrophils (Fig. 2b,i) were computed using the Plot Profile function in FIJI. Specifically, rectangualr ROI’s spanning the length of the cell (mpx:LifeAct-mRuby) were manually drawn. Mean intensities (averaged over ROI width) were computed to generate vectors of variable length corresponding to individual cells. These raw data vectors were then re-binned over ten bins using custom MATLAB code, and intensity values normalized to maximum intensity value for each cell. The ratio of central to leading edge LifeAct intensity (Fig. 2e, Fig 3b,f, Extended Data Fig. 8c) was measured by manually tracing an ROI around the middle and front third of neutrophils expressing LifeAct-mRuby during motility within the epithelium or sub-epithelium. Epithelial deformations (Fig 3c,g, Extended Data Fig. 8e) were measured manually in FIJI as the maximum width between epithelial cells during neutrophil passage events.

### Laser wounding to elicit tissue fracturing

Laser ablation of basal keratinocyte tissue at 3 days post fertilization was performed with an Optimicroscan UV (OMS-uv) system equipped with a raster scanning point stimulation illuminator with a 1KHz pulsed 355nm laser and another optical port for visible light photo-stimulation (405nm). A pair of galvos directed the laser to a region of interest, defined by the user in NIS Elements. A circular region of interest of 30–40 μm diameter was defined in a single focal plane. Immediately following ablation, images were acquired at 10–15 second intervals, on the Nikon Ti2 confocal spinning disk microscope with the 40X objective (see above for details).

#### Neutrophil-like PLB-985 cell line maintenance

A PLB-985 cell line stably expressing LifeAct-mRuby, generated by lentiviral transfection as previously described (Schrope et al), was maintained in R02 media, defined as RMPI 1640 media (Thermo Fisher Scientific,11875093) supplemented with 2% fetal bovine serum (FBS; Thermo Fisher Scientific, 10437010) and 1% penicillin/streptomycin at 37 °C and 5% CO2, until differentiation. PLB-985 cells were differentiated into neutrophil-like cells by treatment with 1.25% DMSO (Sigma-Aldrich, D2650) for 6 days at 37 °C and 5% CO_2_ in R02 media.

### Confined migration assay within liquid walled microchannels

Liquid-walled microchannels were constructed as previously described^18,42,43^. Briefly, a chambered cover glass (no. 1.5 borosilicate glass, 0.13 to 0.17 mm thick; Thermo Fisher Scientific, 155360) was grafted with liquid PDMS-silane by chemical vapor deposition then masked with a PDMS stamp and treated with O_2_ plasma at 60 W for 3 min to pattern the surface with exclusive liquid repellent (ELR) regions either aqueous repellent (oil-in-aqueous ELR), or oil repellant (oil-in-aqueous ELR), when overlaid with silicon oil (5 cSt; Sigma-Aldrich, 317667). Liquid-walled (aqueous-oil interface) microchannels were constructed by sweeping aqueous media (R02) containing rat tail collagen I (corning, 3.33 mg/mL final concentration) across the patterned surface with a wide orifice pipette, and allowed to polymerize at pH 7.2 at 37° as previously described^18^. Neutrophil-like PLB-985 cells were added to the inlet droplet at a density of 50,000 cells/uL by pipette (1.5 μL total volume) and 1.5 μL of 100 nM fMLP (Millipore Sigma F3506) added to the outlet droplet. The cell suspension was mixed via pipette to ensure homogeneity and cell positioning near the channel entrance. Devices were incubated at 37° and 5% CO2 for 10–20 minutes to allow cells to enter the migration channel, at which point devices were transferred to spinning-disk confocal (CSU-X; Yokogawa) Zeiss Observer Z.1 inverted microscope equipped with an electron-multiplying charge-coupled device Evolve 512 camera (Photometrics). Images were acquired with an EC Plan-Neofluar 40×/NA 0.75 air objective (1–2μm optical sections, 2,355 × 512 resolution), running ZenPro 2012 software (Zeiss).

### WASP Morpholino

Morpholino oligonucleotides (Gene Tools), stored at room temperature in Danieau buffer (58 mM NaCl, 0.7 mM KCl, 0.4 mM MgSO4, 0.6 mM Ca(NO3)2, 5.0 mM HEPES pH 7.1–7.3), were injected (3 nl) into the yolk of embryos at the single cell stage. Previously validated morpholino targeting WASP1 (5′−5′-GCCCTTTGCTTTTGCCTTTGCTCAT-3′−3′)^20,21^ and standard control morpholino (5′-CCTCTTACCTCAGTTACAATTTATA-3′; 300 μM) was injected at concentrations of 300 μM. Injected embryos were incubated in E3 medium at 28.5°C until imaging at three days post fertilization.

### Pharmacological inhibitors

The following small molecule inhibitors were used at the listed final concentrations: ZCL278 (50 μm, Cayman chemical 14849) to inhibit Cdc42^44^, Cytochalasin D (5 μM, Sigma Aldrich C8273) to inhibit actin polymerization, and CK666 (10 μm, Millipore Sigma, SML0006) to inhibit ARP2/3. All inhibitors were reconstituted and stored as instructed by the vendor. Larval fish were treated in inhibitors at the above specified concentration in E3 media for 1 hour at 28.5° prior to imaging.

### Generation of itgae.2 mutants

The protocol for generating *itgae.2* mutants was based on previously outlined methods^45^, with specific details provided in Supplemental Figure 8a-b. Synthego’s CRISPR Design Tool was used to predict two gRNAs for the itgae.2 gene, which were used as a pair, targeting both Exon 2 and Exon 16. For each gene, an injection mix was prepared containing two gRNAs (final concentration of 10uM each) and purified Cas9 protein (final concentration of 20uM, Synthego). Wildtype embryos (AB) were microinjected with 2 nl of the injection mix at the 1-cell stage. Imaging experiments were performed at 3 dpf, immediately after which genomic DNA was extracted from the injected larvae and subjected to genotyping PCR to validate efficacy of the gRNA’s for each individual fish. Primers used in genotyping *itgae.2* were as follows: Exon 2 forward, 5′-TATTGTGCAATGCAACCG-3′; Exon 2 reverse, 5′-CCATCTTTTGACTGAATG-3′; Exon 16 forward, 5′-GAGGACAGGTTTGAACGT-3′; Exon 16 reverse, 5′-AGCTGAGCCTGTTTCCAG-3′. A representative example of the result is shown in Supplemental Figure 8a-b. Injections of gRNA targeting tyrosinase were used as a control.

### Tracking E-cadherin cluster displacements

E-cadherin cluster displacements were tracked using the Manual Tracking plugIn in FIJI from maximum intensity z-projections of confocal z-stacks at 1 μm step size. Time-projection plots (Fig. 4d) and kymographs (Fig. 4e) were generated using the Temporal Color Code and Generate Kymograph commands. E-cad cluster velocities (Fig. 4f) were calculated from the slope of kymograph lines, representing displacement (μm) over time (secs). Angles of E-cad cluster displacement relative to cell direction were quantified using the Angle Tool in FIJI to measure the angle between the E-cad cluster at its maximally displaced point in reference to the nearest cell membrane point along the axis of migration.

### Dynamics and morphology of LifeAct foci at the cell front

LifeAct foci were identified using the built in Automated Local Thresholding function in FIJI (Fig. 4g, Fig. 5). The area of LifeAct foci (Fig. 5c) were then measured by manual ROI tracing, and co-localisation with E-cadherin signal (Fig. 5e) measured as the mean signal intensity of E-cad:YFP signal overlapping with the freehand ROI defining the LifeAct foci. Foci positioning, defined as distance from the front of the cell (Fig. 5d), was measured manually using the Line Tool in FIJI.

### Zebrahub data visualization

Processed Zebrahub transcriptome data object (zf_atlas_full_v4_release.h5ad) was downloaded (https://zebrahub.ds.czbiohub.org/data) and converted into the H5Seurat format for analysis in the R environment with Seurat (v.4.3.0)^46^. Cells from all developmental stages (10 hpf - 10 dpf) were included to show gene expression distribution using *FeaturePlot*.

### Statistical Analysis

Statistical significance was determined using Prism 10.0; GraphPad Software, applying student’s *t* tests assuming equal variance. All plots depict data acquired over three independent experimental replicates, either distinct zebrafish spawning events or PLB-985 cell differentiations.

## Supplementary Material

Supplementary Files

This is a list of supplementary files associated with this preprint. Click to download.

- SupplementaryVideo5.avi
- SupplementaryVideo4.avi
- SupplementaryVideo6.avi
- SupplementaryVideo7.avi
- SupplementaryVideo2.avi
- SupplementaryVideo1.mp4
- extendeddata.pdf
- SupplementaryVideo3.avi

**Supplementary Video 1:** 3D rendering of z-stack confocal images representing neutrophils (mpx:mCherry) in the epithelial layer (pseudocolored magenta) and sub-epithelium (pseudocolored blue), generated in Imaris 11. Basal keratinocytes labeled in green (krtt1c19e:acGFP).

**Supplementary Video 2:** Z-projected, timelapse depiction of neutrophil (mpx:mCherry) migration through epithelial maze (krtt1c19e:acGFP).

**Supplementary Video 3:** Z-projected, timelapse depiction of nuclear positioning during neutrophil passage between surrounding epithelial cells. The neutrophil nucleus (LyzC:H2B-mCherry) is pseudocolored grey and the neutrophil cytoplasm (lyzC:BFP) pseudocolored magenta for better visualization. Basal keratinocytes (krtt1c19e:acGFP) are labeled in green.

**Supplementary Video 4:** Z-projected, timelapse depiction of neutrophil (mpx:mCherry) generation of expansile forces to deform surrounding epithelial cells (krtt1c19e:acGFP).

**Supplementary Video 5:** Z-projected, timelapse depiction of neutrophil actin dynamics (mpx:LifeAct-mRuby) under treatment with DMSO control (left), Cytochalasin D (5 μM) to inhibit actin polymerization (middle), or CK666 to inhibit ARP2/3 (10 μM, right). Both inhibitors result in loss of both leading edge and centralized actin, and impair motility through the epithelium (krtt1c19e:acGFP, pseudocolored grey).

**Supplementary Video 6:** Z-projected, timelapse depiction of epithelial cadherin (E-cad) cluster dynamics (Cdh1:Cdh1-YFP, pseudocolored grey). Clusters exhibit minimal displacement over ten minutes (frame interval of 15 seconds).

**Supplementary Video 7:** Z-projected, timelapse depiction of neutrophil F-actin (mpx:LifeAct-mRuby, left) and E-cad cluster (cdh1:cdh1-YFP, middle) dynamics during intra-epithelial neutrophil passage (merged, right). White arrow indicates examples of E-cad clusters moving retrograde to cell direction, then relaxation following neutrophil passage.

## Data Availability

All data are available in the main text or the supplementary materials, or upon request.
